# Revisiting the role of computational neuroimaging in the era of integrative neuroscience

**DOI:** 10.1038/s41386-024-01946-8

**Published:** 2024-09-06

**Authors:** Alisa M. Loosen, Ayaka Kato, Xiaosi Gu

**Affiliations:** 1 Department of Psychiatry, Icahn School of Medicine at Mount Sinai, New York, NY USA; 2 Center for Computational Psychiatry, Icahn School of Medicine at Mount Sinai, New York, NY USA; 3 Nash Family Department of Neuroscience, Icahn School of Medicine at Mount Sinai, New York, NY USA

**Keywords:** Cognitive neuroscience, Translational research

## Abstract

Computational models have become integral to human neuroimaging research, providing both mechanistic insights and predictive tools for human cognition and behavior. However, concerns persist regarding the ecological validity of lab-based neuroimaging studies and whether their spatiotemporal resolution is not sufficient for capturing neural dynamics. This review aims to re-examine the utility of computational neuroimaging, particularly in light of the growing prominence of alternative neuroscientific methods and the growing emphasis on more naturalistic behaviors and paradigms. Specifically, we will explore how computational modeling can both enhance the analysis of high-dimensional imaging datasets and, conversely, how neuroimaging, in conjunction with other data modalities, can inform computational models through the lens of neurobiological plausibility. Collectively, this evidence suggests that neuroimaging remains critical for human neuroscience research, and when enhanced by computational models, imaging can serve an important role in bridging levels of analysis and understanding. We conclude by proposing key directions for future research, emphasizing the development of standardized paradigms and the integrative use of computational modeling across neuroimaging techniques.

## Introduction

Neuroimaging has been a cornerstone of human cognitive neuroscience and mental health research for decades, significantly advancing our understanding of the brain mechanisms underlying cognition, behavior, and their alterations in psychiatric and neurological disorders (e.g., [1–3]). The most widely used neuroimaging techniques include *functional magnetic resonance imaging* (fMRI; cf. Box 1 for a glossary of all italicized terms), *magnetoencephalography* (MEG), and *electroencephalography* (EEG). These methods have revealed important insights into the neural mechanisms of a wide range of mental functions, from sensorimotor processes to emotion and memory to beliefs and social cognition (e.g., for review see [4–10]).

In recent years, computational models have played an increasingly prominent role in human neuroimaging studies. By simulating neurobiological processes and linking observed data to underlying mechanisms, these models offer a framework for decoding complex brain-behavior relationships and predicting cognitive, behavioral, and clinical outcomes. The emergence and evolution of *computational neuroimaging* have thus generated widespread enthusiasm for their potential to significantly enhance our understanding of brain function and improve how we characterize and predict clinical conditions (e.g., [11]). While this enthusiasm has not completely faded, the limitations of neuroimaging have also become clearer, especially in the context of research on higher-order cognition and psychiatric disorders (e.g., [1, 12–14]).

Moreover, emerging tools driven by rapid neuro-technological advancements are competing for the spotlight that neuroimaging has long enjoyed. Recent developments such as invasive recordings of human brain activity (e.g., [15–17]) and real-time and real-life recordings via wearables (e.g., [18–20]) highlight the known limitations of traditional imaging methods by providing unprecedented access to either neural data of high temporospatial resolution or more ecologically grounded measurements. These advancements significantly broaden the methodological repertoire of the field, offering new avenues for both basic and clinical research.

In this rapidly evolving landscape, a crucial question arises: Will neuroimaging fade as a field in human neuroscience research, even with the aid of computational models? This review seeks to address this question by offering a comprehensive perspective, as, to our knowledge, no existing publication has fully examined the dynamic interplay between computational and neuroimaging techniques compared to other emerging methods in human neuroscience.

We will critically re-evaluate neuroimaging’s role within this shifting methodological landscape. Beginning with an overview of the current state of computational neuroimaging, we will emphasize the reciprocal relationship between computational approaches and neuroimaging. Next, we will assess the strengths and limitations of computational neuroimaging by comparing it with alternative methods, ranging from invasive intracranial recordings to non-invasive wearable devices. Finally, we will provide a forward-looking perspective, highlighting the importance of integrating diverse methodologies and a solid understanding of neurobiological mechanisms to advance both fundamental research and clinical applications in neuroscience.

Box 1 Glossary **Black box problem:** Refers to the challenge of understanding the internal workings of the brain, even when inputs (stimuli) and outputs (behavior or cognition) are known.

**Computational neuroimaging**: A key area in cognitive neuroscience and mental health research. Computational neuroimaging combines computational models with advanced imaging techniques (e.g., fMRI, MEG, EEG; cf. below) to examine the brain’s role in cognition, behavior, and mental-health conditions. It employs mechanistic and predictive models, which link neuroimaging signals to brain functions and predict outcomes based on acquired data respectively.

**Convolutional neural network (CNN):** A deep learning method usually used to analyze visual data. CNNs are particularly suited for recognize patterns within images (e.g., textures, shapes).

**Data-driven models**: Computational models derived primarily from analysis of large-scale neural or behavioral datasets. These models aim to identify patterns, correlations, or statistical relationships within data.

**Deep brain stimulation (DBS):** A neurosurgical procedure involving the implantation of electrodes within specific brain regions that deliver controlled electrical impulses to modulate brain activity. Open-loop devices are the most common type of DBS; stimulation settings are pre-determined and do not change in response to the patient’s state. Closed-loop devices are emerging and less common. They use sensors to monitor brain activity in real-time, and stimulation settings are adapted automatically based on brain activity linked to symptoms.

**Ecological validity:** The degree to which experimental findings accurately reflect or predict real-world behaviors and cognitive processes. High ecological validity indicates that results from a study are likely to generalize to everyday situations.

**Electrocortical electrogram (ECoG)**: An invasive electrophysiological monitoring technique that uses an electrode grid placed directly on the exposed surface of the brain to record electrical activity from the cerebral cortex.

**Electroencephalography (EEG)**: Scalp EEG is a non-invasive neuroimaging technique that measures electrical brain activity using scalp sensors. In comparison to other non-invasive methods, EEG has a relatively good temporal but a lower spatial resolution making it better for time-sensitive tasks (e.g., identification of rapidly presented stimuli).

**Functional magnetic resonance imaging (fMRI)**: A non-invasive neuroimaging technique that measures brain activity by detecting changes associated with blood flow (i.e. BOLD signal). In comparison to other non-invasive methods, fMRI has a relatively good spatial but lower temporal resolution, meaning it enables researchers to map the functional areas of the brain during tasks or rest.

**Intracranial recordings**: An invasive method involving the placement of electrodes directly onto the surface of the brain or within brain tissue to record electrical activity. This technique is used in research and clinical settings to study brain functions and to localize epileptic seizures.

**Lasso regression:** A form of regularization for linear regression models. Regularization is a statistical method used to reduce errors caused by overfitting training data.

**Machine learning (ML):** A field of artificial intelligence where computer algorithms learn to perform tasks or make predictions [24]. ML algorithms improve their performance through exposure to data which is called “training”. Supervised learning refers to algorithms that learn from labeled training data (e.g., training a model to classify neuroimaging data based on known psychiatric disorder). Unsupervised learning refers to algorithms that identify patterns and structures within unlabeled data (e.g., clustering neuroimaging data to identify subgroups based on shared structural or functional characteristics).

**Magnetoencephalography (MEG)**: A non-invasive imaging technique that measures the magnetic fields produced by neural activity in the brain. Compared to other non-invasive methods, MEG has a medium-level spatial and temporal resolution. Consequently, MEG is used to study a wide-range of brain functions, such as sensory processing and cognitive tasks.

**Mechanistic models**: Theory-driven computational models that leverage existing knowledge to gain insights into the mechanisms underlying processes and observations. In neuroimaging, they operate on the premise that signals reflect brain mechanisms and neurobiology, thereby predicting cognitive behaviors, which facilitates empirical validation.

**MEG-OPM**: A novel neuroimaging technique that measures magnetic fields generated by brain activity. MEG-OPM uses highly sensitive Optically Pumped Magnetometer (OPM) sensors, eliminating the need for cryogenic cooling found in traditional MEG systems. This allows for greater flexibility in sensor placement, potential for wearable systems, and reduced cost.

**Model selection:** The process of selecting the most appropriate model (or set of models) to represent a given dataset or address a specific research question. Relevant methods include cross-validation (dividing data into training and testing sets to evaluate performance on unseen data), information criteria (statistical measures balancing model fit and complexity), and Bayesian model comparison (evaluating the relative probabilities of different models).

**Model simulation:** Model simulations involve running a computational model with specified parameters to generate predicted data and outputs. This allows researchers to investigate the model’s behavior under different conditions and generate hypotheses that can be tested against behavioral or neuroimaging data.

**Paradigm design:** The process of creating experimental tasks and procedures to investigate specific neurological, cognitive, or behavioral processes. A paradigm outlines the stimuli presented to participants, the responses they are expected to make, and the instructions given to guide their behavior.

**Parameter estimation:** The process of determining the numerical values of parameters within a computational model. These parameters represent variables that influence the model’s output, such as learning rates in RL models.

**Philosophy of science:** A branch of philosophy dedicated to addressing fundamental questions related to what science is, how it works, and the logic through which we build scientific knowledge and theories.

**Predictive models**: Computational models that are mechanistic and theory-agnostic but data-driven and used to forecast future observations or infer states based on acquired data [24]. In neuroscience, predictive models are used to predict behavioral outcomes, treatment response, or group memberships (e.g., patient versus no-patient) based on neuroimaging, behavioral or even genetic data.

**Psychometric properties:** Here, psychometric properties refer to the statistical qualities of psychological tests or cognitive-behavioral task measurements. Key psychometric properties are internal validity and test-retest reliability. Internal validity refers to the degree to which a measure taken from one part of e.g., a cognitive-behavioral task, corresponds to the same measures taken from another part of the same task. Test-retest reliability refers to the consistency of measures across multiple administrations.

**Reinforcement learning (RL)**: A type of machine learning where an agent learns to make decisions by taking actions in an environment to maximize cumulative reward [54]. RL is used in neuroscience to model learning and decision-making processes in the brain and has been linked to mechanisms of the dopaminergic system.

**Support vector machines (SVM):** A supervised machine learning method used for classification and regression purposes.

**Theory-driven models**: Computational models built upon established neuroscientific theories and principles, aiming to simulate and predict specific brain functions or behaviors.

**Validation:** The process of evaluating how well a computational model represents the real-world system or phenomenon it aims to simulate. This involves assessing whether the model’s predictions align with empirical data, behavioral observations, or established (e.g., biological) knowledge.

**Wearables**: Devices that are worn on the body and can measure various physiological and biological markers, including heart rate, activity levels, and sometimes even brain activity. Data from wearables are promising in terms of their ecological validity, as they can be used for continuous monitoring and data collection in real-world settings.

## The current landscape of computational neuroimaging

As methodological developments continue, an increasing number of computational models are being deployed in neuroimaging and neuroscience studies. These models vary not only in their complexity but also in their overall objectives, ranging from providing mechanistic explanations to serving as predictive tools. To understand their distinct purposes and choose the appropriate model for a particular study, we must always bear in mind the basic principles of philosophy of science, which should guide how we identify research questions, generate hypotheses, and interpret findings. These principles are especially important in an era where hypothesis-free, data mining approaches are gaining increasing traction, which are distinct from more classical theory- and hypothesis-driven models designed to examine specific neural and computational mechanisms.

Typically, *theory-driven models* in computational neuroimaging seek to identify the neural computations underlying specific behaviors and mental processes (cf. [ref] for review [21]). These models are grounded in theoretical frameworks, incorporating current knowledge of brain structure, function, and cellular and molecular mechanisms (e.g., dopaminergic activity [22]), to make specific, testable predictions about neural and behavioral outcomes (cf. *Biophysical Models* below). These predictions can then be empirically tested against observed data through simulations and model fitting. Furthermore, variations in the models’ architecture and parameters may provide insights into the neural bases of psychiatric disorders.

In contrast, *data-driven models* adopt a largely theory-agnostic stance, focusing on data mining for pragmatic purposes such as identifying novel patterns in the data or predicting clinical outcomes. Central to the current wave of the artificial intelligence (AI) revolution, these models utilize *machine learning* (ML) techniques (cf. below), including both supervised and unsupervised learning methods ([23]; cf. *Machine Learning Models* below), to achieve outcomes that have pragmatic utility [24–26]. These models prioritize the discovery of empirical relationships within large datasets, often with minimal reliance on existing theories. They offer significant clinical potential through the possible identification of biomarkers or the customization of treatment protocols (e.g., [1, 12, 13, 27]).

More recently, theory-driven and data-driven approaches have also been used in tandem, where “hybrid” models incorporate varying degrees of theoretical assumptions and empirical data [28, 29]. Such work may use theoretical knowledge to guide the selection of features or to interpret the results of data-driven analyses, or they may use empirical data to refine and update theoretical models. The degree to which theory and data are integrated can vary depending on the specific research question, the available data, and the goals of the study.

Different imaging modalities may be better suited for informing computational models of varying scales. For instance, fMRI, with its ability to provide insights into brain activity and localization by detecting changes associated with blood flow, can be used in combination with both theory-driven models that map cognitive functions to specific brain regions and circuits (e.g., [30–32]) and data-driven models that decode mental states from localized brain activity patterns (e.g., [33–35]). EEG, offering higher temporal resolution, lends itself for mechanistic, theory-driven (e.g., [36–38]) and *predictive models* investigating temporally sensitive phenomena (e.g., [39–41]), while also being particularly valuable for inaccessible populations or settings due to its portability and ease of use. MEG, with its combination of relatively high temporal and spatial resolution, can be used for models that require precise timing and localization of neural events (e.g., [42–44]) even when engaging in predictive modeling (e.g., [45–49]). To illustrate these different approaches to computational modeling, we will examine some model types below.

### Reinforcement learning models

Typically used in a theory-driven way, reinforcement learning (RL) models have proven to be a powerful tool in cognitive research, offering insights into both behavioral patterns and neural mechanisms supporting learning and decision-making. Primarily mechanistic, RL models assume agents learn by updating their predictions about outcomes based on the rewards and punishments they have encountered and can build simple as well as more complex learning strategies [50]. A core concept in RL models is the *prediction error* (PE), which quantifies the discrepancy between expected and actual outcomes. This concept has found strong support in neurophysiological studies, with dopaminergic neurons exhibiting firing patterns that closely resemble PEs [22].

RL-inspired computational neuroimaging research has identified mesolimbic structures such as the ventral striatum and frontal cortex (e.g., [51–54]) as key players in reward anticipation and processing. More recent studies have expanded on these findings, exploring alternative learning strategies such as model-based planning (e.g., [55–58]) and the interaction among different decision-making policies (e.g., [59–61]). Furthermore, research has shown that dopaminergic PEs can track detailed reward characteristics such as reward variability [62] and cached or inferred values [63]. This underscores their nuanced and multifaceted roles in the brain as they extend their function to complex scenarios such as social decision-making and learning (e.g., [64–66]).

Crucially, these neural substrates are often disrupted in psychiatric conditions, and computational neuroimaging offers valuable insights into these alterations. RL-based neuroimaging has shed light on how reward and error processing are affected in psychiatric disorders. Examples include attenuated PEs linked to depression (e.g., [67–69]; although not ubiquitously so [70]), heightened error processing associated with obsessive-compulsive disorder (OCD; e.g., [71, 72], and alterations in PEs playing a key role in psychosis (e.g., [73–77]) and addiction (e.g., [78, 79]). These findings demonstrate the potential of RL models, with their basis in neural activity, to capture diverse neurocognitive profiles across different psychiatric disorders, providing valuable insights for both understanding and potentially treating these disorders.

### Bayesian models

Bayesian models offer an alternative and complementary framework to understand fundamental cognitive functions. Traditionally mechanistic, originating in perception theories [80], they use Bayesian statistics to model how the brain updates beliefs based on new evidence [81]. This process involves integrating sensory inputs, viewed as probabilistic evidence from the external environment, with existing knowledge. The brain processes these inputs as uncertain data and updates its beliefs by combining prior knowledge with newly perceived information.

Computational neuroimaging with Bayesian models has advanced our understanding of how sensory information is integrated (e.g., [82–85]) and aided investigations into higher-level cognition such as self-evaluation (e.g., [86–88]), and beliefs about the changing external world (e.g., [89–92]). While most often used to characterize behavior, these models, by incorporating principles grounded in neural activity, offer a unique perspective on the neural mechanisms that may underlie these cognitive processes. Bayesian approaches have, thereby, offered insights into how disrupted belief updating can contribute to perceptual disturbances characteristic of conditions such as psychosis (e.g., [93, 94]). Moreover, by quantifying individual differences in belief updating, these models may be used to aid classification and, therefore, data-driven, predictive purposes as well.

### Biophysical models

Biophysical models are a typical form of theory-driven models that explicitly simulate the biological and physiological properties and mechanisms of the brain [14, 95, 96]. They aim to bridge the gap between theoretical constructs and actual brain activity by incorporating detailed knowledge about neural mechanisms at various levels, from single neurons and neurotransmitters to brain regions and networks. The models thus vary in complexity, with the most complex ones often being difficult to estimate [97].

Biophysical models offer profound insights into how complex activity patterns displayed in e.g., fMRI BOLD signals can emerge from underlying neural interactions and thus support cognition and behavior (e.g., [30, 42, 98]). They are particularly useful in understanding how synaptic-level or neurotransmitter-level alterations, such as those seen in schizophrenia, can lead to widespread brain network dysfunction (e.g., [99, 100]). This ability to link biological processes to system-wide impacts has also proven valuable in investigating how specific medications affect brain function and potentially impact higher-level cognition such as working memory (e.g., [100, 101]).

### Machine learning models

Primarily data-driven, ML models are algorithms capable of learning patterns from data via “training” [23]. However, while typically focusing on identifying empirical relationships within data, some ML models may incorporate elements of mechanistic knowledge to enhance their interpretability and performance.

ML models can be separated into supervised learning, which involves models that learn: from labeled datasets, and unsupervised learning, which focuses on finding patterns in unlabeled data, offering differing functions to the field. Both categories have proven to be helpful tools in cognitive and mental health research. Supervised approaches have shown that neuroimaging data holds predictive information about perceptive experiences (e.g., [49, 102, 103]) as well as higher cognitive variables, associated with decision-making (e.g., [45, 104, 105]) and learning and memory (e.g., [46, 48, 106–108]). Unsupervised methods rely on large datasets, and their popularity in cognitive and mental health research is rising due to the growth of large-scale, (partially) open-source neuroimaging datasets (cf. below).

ML models also span a spectrum of complexity, from simple linear methods such as *lasso regressions*, which can identify predictive features while promoting sparsity, to *support vector machines* (SVMs) for classification [23], and random forests for ensemble learning (e.g., [109, 110]). Additionally, deep learning techniques, particularly *convolutional neural networks* (CNNs; [23]), have gained prominence due to their ability to efficiently handle more complex and richer data from raw neuroimaging data, such as fMRI (e.g., [111–114]) or structural MRI scans (e.g., [115–117]).

Datasets, such as the UK Biobank (requires application and ethical approval; [118]), Alzheimer’s Disease Neuroimaging Initiative (ADNI; available to qualified researchers; [119]), Human Connectome Project (HCP; fully open; [120]), and Adolescent Brain Cognitive Development (ABCD) Study (fully open; [121]), offer researchers vast repositories of brain imaging data, making it possible to identify groups or clusters within the data based on neuroimaging features (e.g., [122, 123]). ML-based neuroimaging has, therefore, significant clinical potential. Studies have identified brain activity patterns that distinguish between healthy individuals and those with (e.g., [124, 125]), or at risk of psychiatric or neurological disorders (e.g., [126]). Additionally, ML models have also been explored to predict treatment outcomes (e.g., [127, 128]). While certain weaknesses still exist (such as the *“black box”* problem or limited interpretability), advancements in ML can lead to more informed treatment allocation, diagnoses and even individualized approaches in psychiatry [1, 129].

It is important to note that the discussed model categories are not mutually exclusive. Many models incorporate elements from multiple approaches, and model types can be combined. For example, some data-driven ML models may integrate mechanistic knowledge to enhance predictive accuracy (e.g., [25, 130, 131]). Within theory-driven studies, Bayesian models and RL can also complement each other or be combined (e.g., [132,133]). RL inherently updates beliefs by integrating prior knowledge (e.g., predicted reward) with new evidence (actual reward), effectively operating within a Bayesian framework. This synergy allows Bayesian methods to optimize RL algorithms, highlighting their potential for integration rather than competition.

Regardless of their specific goals, the extent to which they incorporate biological or mechanistic details, or whether they are theory-driven or data-driven, all computational models in neuroimaging rely on high-quality datasets. These datasets are essential for robust parameter estimation, validation, and generalization of findings, facilitating the creation of more accurate and biologically plausible models, while also influencing the choice of model and the degree to which theory-driven or data-driven approaches are emphasized. Rich high-quality datasets can even facilitate the development of more complex and nuanced models. As such, the relationship between empirical data and models, further explored in the next sections of this paper, is truly bidirectional.

## A reciprocal relationship between neuroimaging and computational modeling

As outlined above, computational neuroimaging continues to revolutionize our understanding of the brain across diverse domains. Despite the ever-evolving landscape of methods and data sources, neuroimaging remains fundamental. We argue that its unique data and the knowledge derived from it offer a critical foundation for designing and validating computational models, such as the ones discussed above, with applications extending far beyond those directly involving neuroimaging datasets.

**Fig. 1:**
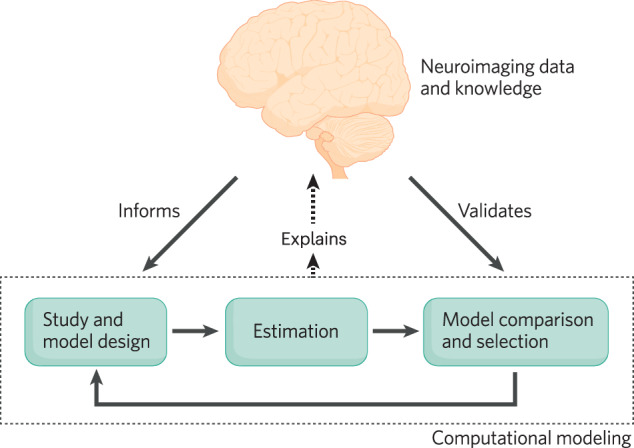
A reciprocal relationship between neuroimaging and computational modeling. Depicted is the cyclical and mutually beneficial relationship between computational modeling and neuroimaging. Neuroimaging insights guide the development of experimental paradigms and computational models (Study & Model Design). These models are then fitted to data and evaluated (Estimation). Model comparison and selection can leverage neuroimaging evidence, enhancing the biological plausibility of the chosen models. The insights gained from this process, in turn, refine future study designs and model development, ultimately advancing our understanding of the brain.

Neuroimaging provides vital insights that can inform each stage of the core workflow of computational studies, which involves *paradigm design*, *simulation*, *parameter estimation*, *model selection*, and *validation* [134, 135]; cf. Fig. 1). When investigating cognitive processes such as learning, memory, and decision-making (even in purely behavioral studies), study design and tasks should be based on established neural mechanisms (e.g., the dopaminergic system’s role in reinforcement as described above). Neuroimaging data can further refine this process by identifying brain regions associated with specific functions. This knowledge helps tailor task complexity (e.g., simple visual search vs. complex decision-making), suggests appropriate model architectures (e.g., hierarchical vs. non-hierarchical), and can even be incorporated directly into simulations to test model assumptions and generate testable predictions. Ideally, model and experiment design interact iteratively [135], guided by neural insights. This ensures the model can effectively analyze the generated data, promoting the development of robust, neuroanatomically-informed hypotheses. Importantly, this integrative approach is valuable even for data-driven models and studies relying solely on simulations, as it grounds the work in established neuroscience.

Consider the multi-armed bandit task, a RL paradigm where an agent must choose between options with unknown reward probabilities to maximize overall rewards (e.g., [136–139]). Neuroimaging findings have been instrumental in shaping both the design of such tasks and the development of corresponding computational models. For instance, extensive evidence implicates dopaminergic brain regions in PE-based learning during the completion of  such and similar learning tasks (e.g., [54, 56, 59, 140]). Furthermore, neuroimaging studies have led to the refinement of existing reinforcement learning models by incorporating parameters that capture certain behaviors with increased precision. For instance, the variability in reward-related brain activity (e.g., in the ventral striatum) can be seen as a motivation of the addition of a ‘reward sensitivity’ parameter to existing RL models (e.g., [138, 139]). In turn, individual differences in reward sensitivity have been linked to psychiatric symptoms and disorders (cf. for review [68, 141]).

Neuroimaging data can also serve as a powerful tool for external model validation that goes beyond internal methods such as cross-validation. By comparing a model’s predictions to independent neuroimaging data, we can directly assess biological plausibility in the context of other relevant data. For instance, Na et al. [66] conducted comparisons of various fMRI general linear models that included parameter estimates from several candidate models of social controllability. They found that the most computationally plausible model (e.g., based on deviance information criteria, DIC) also generated neural activations consistent with existing neuroscientific frameworks (e.g., ventromedial prefrontal cortex tracks model-based learning), as opposed to alternative models that did not generate such activity (in fact, no neural activity). This approach strengthened the model’s plausibility by demonstrating convergence of evidence between model predictions, neural data, and existing knowledge.

In summary, neuroimaging and computational modeling are mutually beneficial to each other, when used strategically and guided by philosophy of science principles. As established, computational models offer a powerful framework for interpreting complex neuroimaging data. However, neuroimaging can, and arguably should, transcend the role of a passive data source and actively inform modeling processes (e.g., [66]). Neuroimaging’s unique advantages in interpretability, accessibility, and clinical relevance make it crucial for validating computational models against real brain activity, enhancing their biological plausibility and external validity. This validation strengthens the link between computational models and real-world brain function, driving advancements in cognitive neuroscience and mental health research. While this interplay can theoretically involve other neural data, neuroimaging’s versatility and established presence in clinical settings make it a uniquely powerful tool in bridging the gap between theoretical models and practical applications.

## Remaining challenges in computational neuroimaging

Despite the promises offered by computational neuroimaging, there are challenges that could impede its progress and may put it at a disadvantage compared to new emerging measures. These limitations include the low ecological validity and questionable psychometric properties of many computational neuroimaging paradigms. Additionally, lab-based neuroimaging techniques and computational modeling each present inherent challenges, such as high costs and strict exclusion criteria for neuroimaging, and limited interpretability for computational modeling. If not properly addressed, these challenges could significantly dampen the future prospects for computational neuroimaging in generating real scientific and clinical value.

*Ecological validity*, which refers to how well findings translate to real-world behaviors and cognition, has been a challenge for computational neuroimaging studies for mainly two reasons. First, the physical setting of neuroimaging environments (e.g., MRI scanners) requires participants to remain completely still in a confined space, which may inhibit the naturalness of their behaviors. Second, computational neuroimaging studies often rely on highly simplified behavioral tasks with many trials (usually hundreds), designed to ensure reliable estimates of specific parameters with as few confounds as possible. While this approach may allow researchers to better separate key cognitive processes, these tasks could be monotonous, not engaging, and lack real-world relevance. Consequently, such paradigms may be problematic especially for measuring high-order cognition (e.g., social behaviors) or studying patients with complex real-life symptoms [142, 143]. For example, individuals with major depressive disorder (MDD) often exhibit altered sensitivity to rewards and punishments in real-life settings. However, laboratory measures relying on simple, monetary decision-making tasks have yielded inconclusive results regarding their RL behaviors, suggesting a need for more advanced methodological approaches to accurately capture the complexities of individual or group-based RL differences [144]. In addition, the inherent complexity of some tasks, such as their high context dependency or temporal dynamics, along with the intricate interaction of multiple task parameters, can further confound the interpretation of results and limit their generalizability to real-world situations.

A second major concern is the lack of consideration of psychometric properties (i.e., internal consistency and test-retest reliability; cf. Glossary) in many existing computational neuroimaging studies [145]. Psychometric robustness is essential for reliable findings in cognitive and mental health research, regardless of whether the study design is cross-sectional or longitudinal. Without it, research might face a replication crisis (as seen before [146]), compromising confidence in findings and slowing down or even jeopardizing clinical translation. Fortunately, the field is recognizing this need for psychometric assessment (e.g., [147–154]) and concrete solutions have been offered (e.g., hierarchical modeling; [152, 155, 156]). However, broader implementation of these good practices is still needed for rigor and reproducibility purposes.

There are many other challenges that permeate all types of neuroimaging and computational research. For example, high equipment costs of neuroimaging devices (e.g., fMRI and MEG) limit access for smaller institutions, potentially hindering research and clinical applications. Additionally, safety concerns associated with certain imaging techniques can restrict participant compatibility, leading to less representative datasets. The indirect nature of brain activity measurements, along with noise introduced during data processing, create challenges for interpreting results and drawing mechanistic conclusions. These challenges are further complicated by the varying temporal and spatial resolutions of different neuroimaging modalities as pointed out below.

Furthermore, computational models, while powerful, can become less useful if their complexity hinders accurate parameter estimation [97]. Predictive models, such as those utilizing ML, may lack sufficient interpretability (cf. *“black box”* problem) or generalizability when training datasets are small or subject to sampling bias (e.g., [27, 145]). Finally, there is a danger of computational models becoming detached from biological reality (cf. [157] for a discussion on biological plausibility in the field). However, this risk can, be mitigated for instance, by actively and continuously integrating neuroimaging knowledge. We will address this integration and the overall promises of new emerging neurological and behavioral measures to the field of cognitive and mental health research in the following section.

## Computational neuroimaging as a bridge: navigating the era of invasive recording, wearables, and digital phenotyping

**Fig. 2:**
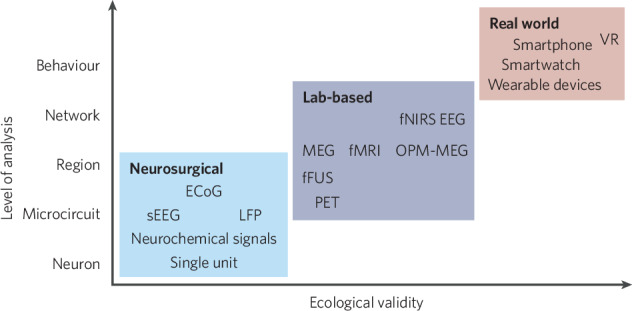
Measurement techniques available for studying neurocognitive functions. Different types of measurements used in 2024 to gain neurological insight are displayed. Measurements are positioned according to their approximate ecological validity (x-axis) and neurological insight (y-axis). Neurons, microcircuits, regions, and networks represent different levels of spatial organization within the brain, from the smallest functional units to large-scale systems. DBS Deep brain stimulation, LFP Local field potentials, sEEG Stereoelectroencephalography, ECoG Electrocorticography, PET Positron Emission Tomography, fFus functional Ultrasound imaging, fMRI functional Magnetic Resonance Imaging, fNIRS functional Near-Infrared Spectroscopy, EEG Electroencephalography, MEG Magnetoencephalography, OPM Optically Pumped Magnetometers, VR Virtual Reality.

Given these challenges, one may question whether neuroimaging will remain a primary method for computational neuroscience and psychiatry studies in humans. Over the years, various measurement techniques for neural activity and behavior have emerged (cf. [158, 159] for review). For example, invasive recordings, non-invasive *wearables*, and smartphones have emerged as powerful tools for human neuroscience research. Each offers unique strengths in terms of temporal and spatial resolution and the ability to capture ecologically valid behavior (cf. Figure 2). Here, we contrast computational neuroimaging with these alternative methods and argue that despite their strengths, neuroimaging remains essential for bridging the gap between different levels of measurement.

### Comparison with intracranial recordings in humans

Intracranial studies in humans have gained significant momentum in recent years, offering unique and complementary direct readouts of neuronal activity at finer temporal and spatial resolution (e.g., single neuron spikes) and enabling causal manipulations (e.g., stimulation; cf. [160, 161] for review). These recordings are typically conducted in patients receiving *deep brain stimulation* (DBS, traditionally for movement disorders but more recently also for psychiatric disorders) or epilepsy monitoring. DBS can target subcortical structures such as the subthalamic nucleus and adjacent areas (cf. [162] for review), allowing the recording of local field potentials, single unit, and even neurochemical signals within a very brief time window (e.g., typically up to 30 min) during surgery. In contrast, recordings from epilepsy patients, such as *electrocortical electrogram* (ECoG) and *stereotactic electroencephalography* (sEEG), provide a more flexible experimental time frame (hours to days) and cover wider brain regions, including the orbitofrontal cortex, temporal cortex, hippocampus, cingulate, and the insular cortex (cf. [163] for review). These methods provide higher spatiotemporal resolution and qualitatively different data than traditional neuroimaging techniques, essential to mechanistic insights and to evaluate whether findings stemming from the vast animal neuroscience literature apply to humans.

Despite their immense potential, invasive methods have their own limitations. The invasive nature itself raises ethical concerns (cf. [164, 165] for a detailed discussion), and most participants under study have neurological alterations at baseline (e.g., altered temporal lobe and hippocampal activity in epilepsy and decreased dopamine levels in Parkinson’s disease), which may limit the interpretation and generalizability of results and findings. While emerging chronically implanted closed-loop deep brain recording and stimulation platforms enable recordings of brain activity in deep brain regions in freely-moving humans (e.g., [166–168]), in most cases, they are still collected intraoperatively (i.e., during DBS surgery) or during inpatient hospitalization (i.e., epilepsy monitoring), which may compromise the ecological validity of assessed behavior, similar to, or even worse than in neuroimaging settings.

Although neuro-tech companies such as Neuralink, Precision, and Synchron are advancing brain-computer interface technologies; the ethical and practical hurdles for invasive recording in healthy individuals will remain significant (e.g., [169, 170]). As such, non-invasive, traditional neuroimaging methods remain important as the majority of healthy volunteers and many patient groups still cannot be studied with invasive techniques. Moving forward, combining these valuable invasive-recording data with non-invasive imaging and behavioral approaches will be critical for researchers to both understand causal neural mechanisms with high spatiotemporal resolutions and to derive non-invasive proxies of intracranial signatures for their use in a broader context.

### Comparison with wearable and portable devices

A second major contender to traditional imaging tools are wearable neural devices, such as wearable EEG, *functional near-infrared spectroscopy* (fNIRS), and their combination (EEG–fNIRS; cf. [171] for review). Their noninvasiveness, cost-effectiveness, portability, and ability to facilitate long-term monitoring have made them widely used in consumer neuroscience (e.g., identifying neural responses related to purchasing behaviors [172]), clinical applications (e.g., epilepsy assessment and treatment [173]) post-stroke recovery monitoring [174]), closed-loop interventions for sleep impairment in mood and anxiety disorders [175]), and studies on brain development (cf. [176] for review). In addition to traditional devices, there is a growing demand for less restrictive, wearable devices for functional imaging of the developing infant brain that offer a higher signal-to-noise ratio than fiber-based and low-density fNIRS measurements. High-density diffuse optical tomography technologies are increasingly popular in meeting this need (e.g., [177, 178, 179]).

Another promising development addressing ecological validity challenges is the Optically Pumped Magnetometers- Magnetoencephalography (*OPM-MEG)*, a portable alternative to cryogenic, superconducting MEG systems [180, 181]. Although experimental indoor environments designed to remove background magnetic fields are required, its greater motion tolerance makes it particularly well-suited for recording brain activity from moving participants during immersive and dynamic virtual reality (VR) experiences (cf. [20] for a discussion of mobile neuroimaging).

Wearable devices, such as smartwatches and physical trackers, offer portability, enabling individuals to monitor physiological data while going about their daily activities and participating in studies. However, these devices present several challenges when used in research settings. For instance, unlike lab-based neuroimaging tools, these wearables are typically not designed for controlled experimental manipulation of variables, which is crucial for investigating causal relationships in scientific studies. Additionally, many commercially available wearable devices have lower recording resolution compared to traditional neuroimaging equipment, often capturing only general physiological data rather than detailed neural activity across specific brain regions. These devices are also susceptible to artifacts caused by motion, sweat, and other environmental factors that are common in real-world settings (see [182] for methodological suggestions on data standardization). Furthermore, managing data storage, sharing, and standardization for wearable devices can be particularly challenging for research environments, especially when data is collected “in the wild”, outside the controlled conditions of a laboratory.

### Comparison with smartphone data

Lastly, smartphones have emerged as a powerful research tool, offering the ability to measure naturalistic behavior (e.g., GPS location) and collect large-scale behavioral data via mobile applications (e.g., [183]). Research-oriented apps designed for data collection offer platforms for questionnaires and cognitive tasks. Examples include: The Great Brain Experiment [184], the Neureka App [185], the Brain Explorer App [186], the Happiness Project (https://thehappinessproject.app/info) and the Social Brain App [187]. Commercially available health management apps, particularly when paired with wearables such as an Apple Watch or a Fitbit, can track physiological measures such as sleep, activity, and heart rate in users’ natural environments (i.e., ecological momentary assessment; [188, 189]). The key advantages of employing smartphones in neuroscience research include the potential for large, diverse samples (i.e., 20,000 people submitted complete data for at least one five-minute task in The Great Brain Experiment [184]), multivariate data sets on individuals and dense, repeated assessments in everyday life [183]. This offers a significant shift from lab-based small-sample neuroimaging studies, allowing for inferences regarding epidemiological causality (e.g., [190, 191]) and predictive analyses (e.g., [192, 193]).

While testing participants outside of the lab with smartphone-based tasks that often have fewer trials might raise concerns about noisier data, recent advances in statistical and computational modeling, such as latent factor and hierarchical analyses, have shown promising routes to mitigate this problem [154]. Clinically, smartphones offer a cost-effective and accessible way to monitor patients’ daily activities longitudinally, potentially aiding in identifying early signs of mental health problems.

However, in this context, a primary limitation is that smartphones, due to their inherent design, mainly record behavioral and cognitive data, lacking direct access to brain or other physiological signals signals. While established brain-behavior relationships (for instance the link between memory-related tasks and hippocampal activity; e.g., [48, 108]) can be leveraged to infer neural correlates of smartphone-measured behaviors, these interpretations should be made with caution. Such interpretations may inspire new research, but their biological basis requires validation through simultaneous measurement of neural activity. In addition, challenges in standardizing the smartphone data collection environment can introduce uncontrollable variability in the data [194, 195], similar to that encountered when using wearables for data collection in naturalistic environments mentioned above. Finally, the use of participants’ private smartphones for data collection raises unique ethical concerns, especially if the research protocol could capture data from other functions or apps (e.g., GPS locations, call history, text messages, social media apps). To address these limitations and maximize the value of smartphone data, it is crucial to 1) validate the same measures in the laboratory setting, ideally in combination with neural measures such as neuroimaging data; and 2) to engage in thorough research ethics discussions early on in study design.

## The future of computational neuroimaging

In the rapidly evolving field of human neuroscience, traditional neuroimaging approaches, such as fMRI, may appear less appealing at first glance. However, we argue that when combined with computational methods, neuroimaging can remain essential for research. Offering a balance of mechanistic insight and ecological validity (cf. Figure 2), neuroimaging effectively bridges the gap between emerging neuro-technologies and real-world applications by informing both.

To fully realize this potential, it is crucial to design and adapt psychometrically sound cognitive-behavioral tasks ensuring they can be administered across a variety of environments, including smartphone/wearable, neuroimaging and intracranial settings. This allows direct comparisons of behaviors and links to neural computations and function at multiple levels (from single neurons to circuits).

The Intra-Extra Dimensional Set Shift (ID/ED) task is one example of a task used successfully in various settings. It measures the ability to shift attention and decision-making flexibly [196]. Some research using this task has shown cognitive inflexibility in patients with OCD across behavioral (e.g., [197, 198]), neuroimaging (e.g., [198–200]) and large-scale online and smartphone-based studies (e.g., [201, 202]). Recently, a DBS- study using this task showed that stimulation of the anteromedial subthalamic nucleus selectively improved cognitive flexibility, while stimulation of the ventral capsule/ventral striatum improved mood symptoms [203]. Another example is the probabilistic instrumental learning task has found wide application in behavioral and neuroimaging studies (e.g., [140, 204]). Importantly, its associated computational model exemplifies how model components (i.e., PEs) can bridge data across settings. Intracranial recordings have refined our understanding of the behavioral and neuroimaging findings here by pinpointing interactions between reward and punishment PEs in the prefrontal and insular cortices, thereby elucidating the neural basis of previously identified learning [205].

Thus, computational models offer a powerful bridge for integrating diverse neuroscientific data across multiple studies. By focusing on specific parameters (e.g., PEs and learning rates) across studies, they can combine the strengths of varied datasets, achieving a convergence of evidence: online data (larger but potentially noisy), controlled laboratory and neuroimaging data (brain-network localisation), and even invasive measures (for fine-grained analysis of dopaminergic activity). To advance personalized mental health using neuroscience, understanding neurobiological mechanisms through systematic research is as crucial as building large neuroimaging databases for machine learning (cf. Section 2). A multimodal mindset should permeate the research process, both within and across studies, to fully leverage the strengths of diverse data sources. As the field evolves, this mindset will also enable seamless integration of new neuroimaging and behavioral methods, maximizing their potential (i.e., new emerging Magnetic Particle Imaging; [206]).

In conclusion, as we enter an era of ecologically valid, naturalistic behavioral data (e.g., via smartphones) and ultra-precise intracranial recordings, computational neuroimaging is uniquely positioned to bridge the gap between these diverse sources, establishing meaningful connections between neural activity, behavior, and theoretical constructs. This integration holds immense promise for addressing pressing questions in developmental neuroscience and mental health research.
